# Genome Editing Weds CRISPR: What Is in It for Phytoremediation?

**DOI:** 10.3390/plants7030051

**Published:** 2018-06-28

**Authors:** Zarrin Basharat, Luís A. B. Novo, Azra Yasmin

**Affiliations:** 1 Microbiology & Biotechnology Research Lab, Department of Environmental Sciences, Fatima Jinnah Women University, Rawalpindi 46000, Pakistan; azrayasmin@fjwu.edu.pk; 2 Jamil-ur-Rahman Center for Genome Research, Dr. Panjwani Center for Molecular Medicine and Drug Research, International Center for Chemical and Biological Sciences, University of Karachi, Karachi 75270, Pakistan; 3 GeoBioTec Research Centre, Department of Geosciences, University of Aveiro, 3810-193 Aveiro, Portugal; 4 Centre of Biotechnology and Fine Chemistry—Associated Laboratory, Faculty of Biotechnology, Catholic University of Portugal, 4169-005 Porto, Portugal

**Keywords:** CRISPR, Cas9, genetic engineering, phytoremediation, phytomining, environmental pollution, Cpf1

## Abstract

The arrival of sequence-specific endonucleases that allow genome editing has shaken the pillars of basic and applied plant biology. Clustered regularly interspaced palindromic repeats (CRISPR) is a revolutionary genome-engineering tool that enables the enhancement of targeted traits in plants. Numerous plants, including energy crops, known for their potential to tolerate, immobilize, and stabilize inorganic and organic pollutants, have already been edited using different CRISPR systems. Moreover, a large array of genes responsible for increased metal tolerance, metal uptake and hyperaccumulation have already been identified. Thus, the CRISPR-mediated genome reprogramming of plants, including its use in gene expression regulation through transcriptional repression or activation (CRISPRi and CRISPRa), could be of paramount importance for phytoremediation. The simplicity, inexpensiveness, and capabilities of this gene editing technique could soon be used to enhance plants and bacteria involved in phytotechnologies, such as phystabilization, phytoextraction, phytomining, phytovolatilization, and bio-energy generation. In this brief viewpoint piece, we posit some of the potential benefits of CRISPR for phytoremediation.

## 1. Overview

The advent of the prokaryotic adaptive immune system, centered on clustered regularly interspaced short palindromic repeats, i.e., CRISPR technology, has breathed new life into genome editing endeavors [1]. CRISPR-Cas9 and the novel CRISPR-Cpf1 have been harnessed for generating knock-outs, targeting transcriptional regulation or making substitutions in the genome [2,3]. These systems are focused on a guide RNA (gRNA), coupled with Cas9, a type II endonuclease from *Streptococcus pyogenes*, or Cpf1, a class II type V endonuclease from *Prevotella* and *Francisella*, in order to facilitate direction to a target site [4]. Substantial progress is increasingly being made in this realm, as tools and methods of CRISPR usage in genome editing continue to expand due to its considerable advantage over competing techniques, like zinc finger nucleases (ZFNs) and transcription activator-like effector nucleases (TALENs) [5,6,7]. However, usage of this method for genome engineering aimed at phytotechnologies is underrepresented, despite being the need of the hour. Here, we highlight the potential of this effective tool for precision genome editing of plants to remediate polluted soils and waters (either by organic or inorganic contaminants).

Phytoremediation is a green, solar energy driven, and low cost technology to mitigate the impact of harmful pollutants, which represents a sustainable alternative to other costly, impracticable and often hazardous physicochemical solutions [8]. Additionally, it helps to refurbish natural habitats and heal the hideous scars of the landscape. Ideally, phytoremediators should feature a fast growth rate, high biomass yield, hardiness, tolerance to elevated metal levels, large root system and the capacity to immobilize and/or uptake significant amounts of contaminants [9]. The latter is a property that is strikingly expressed by hyperaccumulators—plants exhibiting shoot metal concentrations, 1–3 orders of magnitude greater than other plants growing in the same environment [10]. Hyperaccumulation and the aforementioned traits are also critical for phytomining, another plant-based technique, akin to phytoremediation, that aims at the extraction of valuable metals from mine tailings and mineralized/polluted soils to obtain an economic revenue [11]. In this connection, several plant species have been identified as attractive candidates for the purpose of phytoremediation and further enhanced by scientists via transgenics [12]. 

## 2. Genetically Engineered Phytoremediation

Genome editing of plants for phytoremediation using CRISPR systems is an unexplored and yet promising venture to increase the remedial capacity of plants. Genomes of model phytoremediators, *Noccaea caerulescens* (the Cd, Ni, and Zn hyperaccumulator, formerly known as *Thlaspi caerulescens*), *Arabidopsis halleri* (Cd and Zn hyperaccumulator), *Pteris vittata* (As hyperaccumulator), *Hirschfeldia incana* (known for its capacity to withstand and uptake Pb), *Brassica juncea* (the Swiss army knife of phytoremediation) and several other species, have been fully or partially sequenced [13,14,15,16,17]. A few energy crops have also been sequenced [18], and editing their genomes for increased tolerance to pollutants could deliver multiple benefits. Manipulation of genomic sequences of these plants may facilitate the identification and characterization of key genetic determinants in the investigation of phytoremediation processes, like phytoextraction, phytostabilization, phytovolatilization, phytodegradation, or phytodesalination, to name a few. Sequence data information of these plants can be utilized to establish CRISPR systems for phytoremediation by targeted engineering of mechanisms involved in the accumulation, complexation, volatilization, and degradation of pollutants. CRISPR could be used to transfer a desired set of instructions in the plant genome in a candid mode, as it is a programmable, next-generation method for high throughput genetic manipulation, as compared with the low throughput ZFNs and TALENs [19,20]. Moreover, the sequence availability of plant genomes, aided by software tools, bioinformatics-based approaches and the availability of codon-optimized versions of Cas9 for monocots as well as dicots, has opened new avenues for using CRISPR-Cas9 genome editing in a wide variety of plants [21].

Areas of focus for phytoremediation may include the CRISPR-mediated expression of genes to increase the synthesis of metal ligands (such as metallothioneins and phytochelatins), metal transport proteins (from the CDF, HMA, MATE, YSL and ZIP families, to name a few), plant growth hormones (AUXs, CKs, and GAs), and root exudates (particularly LMWOA and siderophores). Since the early 2000s, numerous studies have identified plant and bacterial genes that, upon transfer to the target plants, have generated advantageous effects for phytoremediation. For instance, Arabidopsis and tobacco plants, enhanced with the NAS1 gene (which encodes the enzyme NA synthase), showed greater tolerance towards metals like Cd, Cu, Fe, Mn, Ni, and Zn, and increased the uptake of Mn and Ni [22,23]. The overexpression of metallothioneins encoding-genes (MT_A1_, MT1, and MT2) in poplar, tobacco and Arabidopsis plants has increased their capacity to endure and accumulate Cd, Cu, and Zn [24,25,26]. The expression of the metallothionein gene, MT2b (along with the up/down regulation of genes involved in abscisic acid synthesis and catalysis, respectively), is known to increase the ability of *H. incana* to tolerate and accumulate Pb [16]. The transfer of the genes APS and SMT, responsible for the synthesis of ATP sulfurylase and selenocysteine methyltransferase, respectively, enhanced the tolerance and accumulation of Se in *B. juncea* plants [27]. These and many other genes, which could soon be enhanced via CRISPR-technology, have recently been reviewed by Fasani et al. [28] in a paper about transgenically modified plants reclaiming metal-polluted soils. In this comprehensive work, the authors clearly indicate which genes were transferred and their specific source, as well as the observed effects in the target plant. These effects, which include an increased tolerance to toxic metal levels, enhanced metal uptake capacity, and even metal hyperaccumulation, could be critical to boost phytoremediation. Nonetheless, it should be noted that promoting the accumulation of a given metal through the expression of a specific gene could sometimes trigger hypersensitivity to that element in the target plant, i.e., as metal uptake and the corresponding plant detoxification mechanisms may not be governed by a unique gene, but rather a set of genes, this could lead to plant decay. Arazi et al. [29] reported that the overexpression of a plasma membrane protein (NtCBP4) in transgenic tobacco plants increased Pb accumulation, but also significantly enhanced the plant’s sensitivity to the metal. By the same token, the expression of the MerC gene in Arabidopsis and tobacco increased Hg accumulation 2-fold in relation to wild type plants, but rendered the transgenic plants hypersensitive to Hg [30].

The above-mentioned approach could be extended to organic pollutants, ranging from polycyclic aromatic hydrocarbons (PAHs) and polychlorinated biphenyls (PCBs) to explosives like hexahydro-1,3,5-trinitro-1,3,5-triazine (Royal Demolition Explosive; RDX) and 2,4,6-trinitro-toluene (TNT). Numerous studies have reported genes involved in the detoxification and degradation of organic xenobiotics in plants [31,32]. These data could be used as seed materials for future trials involving the CRISPR-mediated enhancement of plant enzyme systems, responsible for the removal and detoxification of organic contaminants. Transgenic Arabidopsis and rice plants, expressing the genes responsible for the naphthalene dioxygenase system, developed the capacity to tolerate and metabolize naphthalene and phenanthrene [33]. The expression of the gene, BphC.B, by transgenic alfalfa plants, significantly increased their tolerance to PCBs and 2,4-dichlorophenol (2,4-DCP) (individually and combined), as well as their capacity to dissipate and remove PCBs and 2,4-DCP, respectively [34]. Rylott et al. [35] have demonstrated that the transfer of bacterial genes, XplA and XplB, to Arabidopsis plants have allowed them to efficiently remove and detoxify RDX through the cytochrome P450-reductase complex.

**Figure 1:**
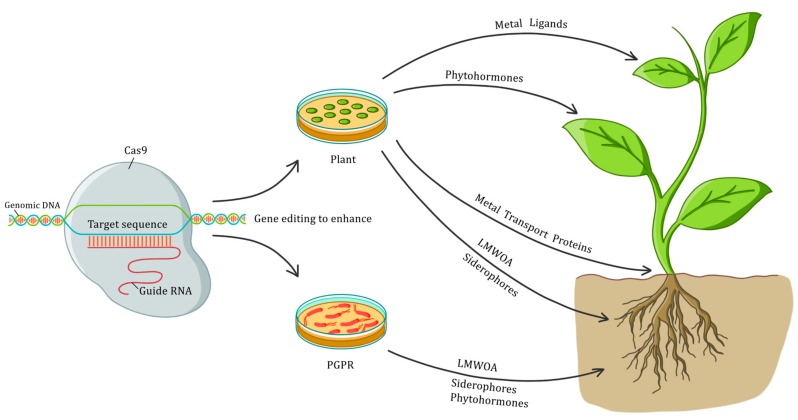
Potential advantages of CRISPR-Cas9 for phytoremediation. Gene editing of plants and plant growth-promoting rhizobacteria (PGPR) with the CRISPR-Cas9 system could increase the synthesis of a number of compounds that are critical to enhance the biomass yield, tolerance to contaminants, and the complexation, transport, accumulation and detoxification of pollutants.

In addition, phytoremediation could also benefit from the application of the CRISPR technology in designing more competent plant growth-promoting rhizobacteria (PGPR) [36]. A growing number of genes with the potential to aid the PGPR-plant interaction, have been reported in the relevant literature. The accounts include genes responsible for the synthesis of phytohormones [37], the nitrogenase complex [38], and siderophores [39], to list but a few. Thus, equipping PGPR with these genes could benefit the ability of plants to grow and handle pollutants by increasing nitrogen fixation, phosphate solubilisation, iron sequestration, phytohormones production (direct mechanisms), and biocontrol (indirect mechanisms) [40]. Altogether, plant and bacterial CRISPR-derived upgrades could take phytoremediation to the next level, allowing the successful reclamation of polluted soils and waters in tractable time frames. Some of the potential pluses of CRISPR-Cas9 for phytoremediation are depicted in Figure 1.

## 3. CRISPR-Mediated Strategies for Futuristic Phytoremediation

The outline of the several strategies that could be tested for their capacity to enhance phytoremediation include the direct transfection of Cas9 along with gRNAs into the plant protoplasts, plant regeneration from single-cells, T-DNA-delivered gRNA–Cas9, modular cloning systems like Golden-Braid or even the cloning free strategy. T-DNA-delivered gRNA-Cas9 (in Agrobacterium mediated T-DNA transformation) has also been tested, but due to the transitory action of T-DNA in callus induction, activity has been observed in somatic tissues via genome integration. To make the most of this strategy, it might be imperative to amalgamate diverse gRNAs with Cas9 in a single T-DNA, as an all-in-one plasmid approach would definitely improve editing [5,41]. Cloning systems, like Golden-Braid, ease the association of pre-made DNA elements with multigene constructs [42,43]. Multiplexed editing regulatory assays employ a cloning-free strategy to ensure the incorporation of a single gRNA in the cells, but effect throughput.

Nevertheless, the segment of CRISPR research that may hold greater potential for phytoremediation is the use of gRNA-guided dCas9 to modulate gene expression. Transcription factors can be fused with dCas9 to repress or enhance transcription by RNA polymerase and, subsequently, upregulate or downregulate the expression of a gene or genes of interest [44]. These techniques, known as CRISPRi (interference) and CRISPRa (activation), can modulate gene expression over a 1000-fold range [45] and have been effectively employed on plants [21,46,47]. Tang et al. [48] have recently demonstrated the ability of CRISPR to reduce Cd accumulation in rice by knocking out the metal transporter gene, OsNramp5. The latter is perhaps the most significant step of CRISPR in phytoremediation to date and highlights the promise of its use in gene transcription regulation. CRISPRi and CRISPRa could soon be used to control the expression of genes, responsible for the production of metal transporters, growth factors, metal solubilizing exudates, or oxidative stress metabolites, in plants and bacteria for phytoremediation purposes.

The CRISPR-Cas9 system has also been successfully employed in the past for genome editing in several plant species of interest (food and energy crops) [18,49]. Owing to complexities in plant genomes, e.g., high ploidy, traditional breeding trials have long turnaround times for each experiment. Furthermore, the low frequency of homologous recombination makes site-specific mutagenesis difficult [18]. There may be a growing need for increased plant biomass, growth rate, disease and climate resistance, metal tolerance, and metal accumulation, as gRNA-Cas9 facilitates the targeting of multiple sequences and, hence, multiple traits simultaneously [5]. In fact, CRISPR-Cas9 systems have already been used to modify the genome of species known for their applicability in phytoremediation processes, poplar and maize [44,50]. Poplar is a recurrent choice in phytoremediation due to its high-biomass production, fast growth, deep and wide root system, distinct adaptability to diverse soils and climates, marked tolerance to organic and inorganic pollutants, and exceptional ability in vegetative reproduction that facilitates its propagation [51]. Likewise, maize is a fast-growing, high biomass yield species with a pronounced metal accumulation capability, and has presented favorable results in phytoremediation and phytomining [52,53]. A recent review by Agarwal et al. [54] summarizes the main breakthroughs in maize genome editing via the CRISPR/Cas9 system to date. More importantly, both poplar and maize have been increasingly used to couple their phytoremediation aptitude with bio-energy generation [55,56], denoting the suitability of CRISPR, not only to improve the process efficiency, but also, on a wider scale, to promote sustainability.

## 4. Perspective

Although CRISPR has shown great promise for genome engineering, results depend on the choice of target site, Cas9/Cpf1 action, design of gRNA, and delivery systems, as well as off-target effects that may impede progress [3,57]. However, we believe that significant breakthroughs will be achieved with time, as our understanding of the system increases. Overall, CRISPR-aided genome engineering heralds great potential for exploiting plant genomes to enhance phytoremediation. Modifying genes of interest, their expression, whole pathway and pollutant homeostasis networks that support hyperaccumulation, tolerance, or degradation, can be revolutionary for cleaning the environment via plants and, concurrently, recovering elements of economic interest and generate energy. We are hopeful that this technique may deliver unparalleled leverage to harness desired traits in one fell swoop, taking phytoremediation to its zenith.

## Abbreviations

AUXs	auxins
Cas9	CRISPR associated protein 9
CDF	cation diffusion facilitator
CKs	cytokinins
Cpf1	CRISPR from *Prevotella* and *Francisella* 1
CRISPR	clustered regularly interspaced short palindromic repeats
dCas9	catalytically inactive Cas9
GAs	gibberellins
gRNA	guide ribonucleic acid
HMA	heavy metal transporting ATPases
LMWOA	low molecular weight organic acids
MATE	multidrug and toxin efflux
MTs	metallothioenins
PCs	phytochelatins
PGPR	plant growth-promoting rhizobacteria
TALENs	transcription activator-like effector nucleases
YSL	yellow strip1-like
ZFNs	zinc finger nucleases
ZIP	zinc-regulated transporter iron-regulated transporter proteins.

## References

[1] Pennisi E. (2013). The CRISPR Craze. Science.

[2] Sander J.D., Joung J.K. (2014). CRISPR-Cas systems for editing, regulating and targeting genomes. Nat. Biotechnol..

[3] Yin K., Gao C., Qiu J. (2017). Progress and prospects in plant genome editing. Nat. Plants.

[4] Zaidi S.S.-A., Mahfouz M.M., Mansoor S. (2017). CRISPR-Cpf1: A New Tool for Plant Genome Editing. Trends Plant Sci..

[5] Bortesi L., Fischer R. (2015). The CRISPR/Cas9 system for plant genome editing and beyond. Biotechnol. Adv..

[6] Ma X., Zhu Q., Chen Y., Liu Y.-G. (2016). CRISPR/Cas9 Platforms for Genome Editing in Plants: Developments and Applications. Mol. Plant.

[7] Tang X., Lowder L.G., Zhang T., Malzahn A.A., Zheng X., Voytas D.F., Zhong Z., Chen Y., Ren Q., Li Q. (2017). A CRISPR–Cpf1 system for efficient genome editing and transcriptional repression in plants. Nat. Plants.

[8] Mendez M.O., Maier R.M. (2008). Phytostabilization of mine tailings in arid and semiarid environments—An emerging remediation technology. Environ. Health Perspect..

[9] Padmavathiamma P.K., Li L.Y. (2007). Phytoremediation Technology: Hyper-accumulation Metals in Plants. Water Air Soil Pollut..

[10] Pollard A.J., Reeves R.D., Baker A.J.M. (2014). Facultative hyperaccumulation of heavy metals and metalloids. Plant Sci..

[11] Novo L.A.B., Castro P.M.L., Alvarenga P., da Silva E.F., Ansari A.A., Gill S.S., Gill R.R., Lanza G., Newman L. (2017). Phytomining of Rare and Valuable Metals. Phytoremediation—Management of Environmental Contaminants, Volume 5.

[12] Cherian S., Oliveira M.M. (2005). Transgenic Plants in Phytoremediation: Recent Advances and New Possibilities. Environ. Sci. Technol..

[13] Briskine R.V., Paape T., Shimizu-Inatsugi R., Nishiyama T., Akama S., Sese J., Shimizu K.K. (2017). Genome assembly and annotation of Arabidopsis halleri, a model for heavy metal hyperaccumulation and evolutionary ecology. Mol. Ecol. Resour..

[14] Xie Q.-E., Yan X.-L., Liao X.-Y., Li X. (2009). The Arsenic Hyperaccumulator Fern Pteris vittata L.. Environ. Sci. Technol..

[15] Mandáková T., Singh V., Krämer U., Lysak M.A. (2015). Genome Structure of the Heavy Metal Hyperaccumulator *Noccaea caerulescens* and Its Stability on Metalliferous and Nonmetalliferous Soils. Plant Physiol..

[16] Auguy F., Fahr M., Moulin P., El Mzibri M., Smouni A., Filali-Maltouf A., Béna G., Doumas P. (2016). Transcriptome Changes in Hirschfeldia incana in Response to Lead Exposure. Front. Plant Sci..

[17] Yang J., Liu D., Wang X., Ji C., Cheng F., Liu B., Hu Z., Chen S., Pental D., Ju Y. (2016). The genome sequence of allopolyploid Brassica juncea and analysis of differential homoeolog gene expression influencing selection. Nat. Genet..

[18] Estrela R., Cate J.H.D. (2016). Energy biotechnology in the CRISPR-Cas9 era. Curr. Opin. Biotechnol..

[19] Jinek M., Chylinski K., Fonfara I., Hauer M., Doudna J.A., Charpentier E. (2012). A Programmable Dual-RNA-Guided DNA Endonuclease in Adaptive Bacterial Immunity. Science.

[20] Mali P., Yang L., Esvelt K.M., Aach J., Guell M., DiCarlo J.E., Norville J.E., Church G.M. (2013). RNA-Guided Human Genome Engineering via Cas9. Science.

[21] Lowder L.G., Zhang D., Baltes N.J., Paul J.W., Tang X., Zheng X., Voytas D.F., Hsieh T.-F., Zhang Y., Qi Y. (2015). A CRISPR/Cas9 Toolbox for Multiplexed Plant Genome Editing and Transcriptional Regulation. Plant Physiol..

[22] Kim S., Takahashi M., Higuchi K., Tsunoda K., Nakanishi H., Yoshimura E., Mori S., Nishizawa N.K. (2005). Increased Nicotianamine Biosynthesis Confers Enhanced Tolerance of High Levels of Metals, in Particular Nickel, to Plants. Plant Cell Physiol..

[23] Pianelli K., Mari S., Marquès L., Lebrun M., Czernic P. (2005). Nicotianamine Over-accumulation Confers Resistance to Nickel in Arabidopsis thaliana. Transgenic Res..

[24] Turchi A., Tamantini I., Camussi A.M., Racchi M.L. (2012). Expression of a metallothionein A1 gene of Pisum sativum in white poplar enhances tolerance and accumulation of zinc and copper. Plant Sci..

[25] Lv Y., Deng X., Quan L., Xia Y., Shen Z. (2013). Metallothioneins BcMT1 and BcMT2 from Brassica campestris enhance tolerance to cadmium and copper and decrease production of reactive oxygen species in Arabidopsis thaliana. Plant Soil.

[26] Xia Y., Qi Y., Yuan Y., Wang G., Cui J., Chen Y., Zhang H., Shen Z. (2012). Overexpression of Elsholtzia haichowensis metallothionein 1 (EhMT1) in tobacco plants enhances copper tolerance and accumulation in root cytoplasm and decreases hydrogen peroxide production. J. Hazard. Mater..

[27] LeDuc D.L., AbdelSamie M., Móntes-Bayon M., Wu C.P., Reisinger S.J., Terry N. (2006). Overexpressing both ATP sulfurylase and selenocysteine methyltransferase enhances selenium phytoremediation traits in Indian mustard. Environ. Pollut..

[28] Fasani E., Manara A., Martini F., Furini A., DalCorso G. (2017). The potential of genetic engineering of plants for the remediation of soils contaminated with heavy metals. Plant. Cell Environ..

[29] Arazi T., Sunkar R., Kaplan B., Fromm H. (1999). A tobacco plasma membrane calmodulin-binding transporter confers Ni^2+^ tolerance and Pb^2+^ hypersensitivity in transgenic plants. Plant J..

[30] Sasaki Y., Hayakawa T., Inoue C., Miyazaki A., Silver S., Kusano T. (2006). Generation of Mercury-Hyperaccumulating Plants through Transgenic Expression of the Bacterial Mercury Membrane Transport Protein MerC. Transgenic Res..

[31] Van Aken B. (2011). Transgenic Plants and Associated Bacteria for Phytoremediation of Organic Pollutants.

[32] Rylott E.L., Johnston E.J., Bruce N.C. (2015). Harnessing microbial gene pools to remediate persistent organic pollutants using genetically modified plants—A viable technology?. J. Exp. Bot..

[33] Peng R.-H., Fu X.-Y., Zhao W., Tian Y.-S., Zhu B., Han H.-J., Xu J., Yao Q.-H. (2014). Phytoremediation of Phenanthrene by Transgenic Plants Transformed with a Naphthalene Dioxygenase System from Pseudomonas. Environ. Sci. Technol..

[34] Wang Y., Ren H., Pan H., Liu J., Zhang L. (2015). Enhanced tolerance and remediation to mixed contaminates of PCBs and 2,4-DCP by transgenic alfalfa plants expressing the 2,3-dihydroxybiphenyl-1,2-dioxygenase. J. Hazard. Mater..

[35] Rylott E.L., Lorenz A., Bruce N.C. (2011). Biodegradation and biotransformation of explosives. Curr. Opin. Biotechnol..

[36] Mosa K.A., Saadoun I., Kumar K., Helmy M., Dhankher O.P. (2016). Potential Biotechnological Strategies for the Cleanup of Heavy Metals and Metalloids. Front. Plant Sci..

[37] Boivin S., Fonouni-Farde C., Frugier F. (2016). How Auxin and Cytokinin Phytohormones Modulate Root Microbe Interactions. Front. Plant Sci..

[38] Chinnaswamy A., Coba de la Peña T., Stoll A., de la Peña Rojo D., Bravo J., Rincón A., Lucas M.M., Pueyo J.J. (2018). A nodule endophytic Bacillus megaterium strain isolated from Medicago polymorpha enhances growth, promotes nodulation by Ensifer medicae and alleviates salt stress in alfalfa plants. Ann. Appl. Biol..

[39] Thode S.K., Rojek E., Kozlowski M., Ahmad R., Haugen P. (2018). Distribution of siderophore gene systems on a Vibrionaceae phylogeny: Database searches, phylogenetic analyses and evolutionary perspectives. PLoS ONE.

[40] Novo L.A.B., Castro P.M.L., Alvarenga P., da Silva E.F. (2018). Plant Growth–Promoting Rhizobacteria-Assisted Phytoremediation of Mine Soils. Bio-Geotechnologies for Mine Site Rehabilitation.

[41] Mikami M., Toki S., Endo M. (2015). Comparison of CRISPR/Cas9 expression constructs for efficient targeted mutagenesis in rice. Plant Mol. Biol..

[42] Liu W., Stewart C.N. (2015). Plant synthetic biology. Trends Plant Sci..

[43] Vazquez-Vilar M., Bernabé-Orts J.M., Fernandez-del-Carmen A., Ziarsolo P., Blanca J., Granell A., Orzaez D. (2016). A modular toolbox for gRNA–Cas9 genome engineering in plants based on the GoldenBraid standard. Plant Methods.

[44] Miglani G.S. (2017). Genome editing in crop improvement: Present scenario and future prospects. J. Crop Improv..

[45] Gilbert L.A., Horlbeck M.A., Adamson B., Villalta J.E., Chen Y., Whitehead E.H., Guimaraes C., Panning B., Ploegh H.L., Bassik M.C. (2014). Genome-Scale CRISPR-Mediated Control of Gene Repression and Activation. Cell.

[46] Piatek A., Ali Z., Baazim H., Li L., Abulfaraj A., Al-Shareef S., Aouida M., Mahfouz M.M. (2015). RNA-guided transcriptional regulation in planta via synthetic dCas9-based transcription factors. Plant Biotechnol. J..

[47] Lowder L.G., Zhou J., Zhang Y., Malzahn A., Zhong Z., Hsieh T.F., Voytas D.F., Zhang Y., Qi Y. (2018). Robust Transcriptional Activation in Plants Using Multiplexed CRISPR-Act2.0 and mTALE-Act Systems. Mol. Plant.

[48] Tang L., Mao B., Li Y., Lv Q., Zhang L., Chen C., He H., Wang W., Zeng X., Shao Y. (2017). Knockout of OsNramp5 using the CRISPR/Cas9 system produces low Cd-accumulating indica rice without compromising yield. Sci. Rep..

[49] Song G., Jia M., Chen K., Kong X., Khattak B., Xie C., Li A., Mao L. (2016). CRISPR/Cas9: A powerful tool for crop genome editing. Crop J..

[50] Fan D., Liu T., Li C., Jiao B., Li S., Hou Y., Luo K. (2015). Efficient CRISPR/Cas9-mediated Targeted Mutagenesis in Populus in the First Generation. Sci. Rep..

[51] Baldantoni D., Cicatelli A., Bellino A., Castiglione S. (2014). Different behaviours in phytoremediation capacity of two heavy metal tolerant poplar clones in relation to iron and other trace elements. J. Environ. Manag..

[52] Anderson C., Moreno F., Meech J. (2005). A field demonstration of gold phytoextraction technology. Miner. Eng..

[53] Ali H., Khan E., Sajad M.A. (2013). Phytoremediation of heavy metals—Concepts and applications. Chemosphere.

[54] Agarwal A., Yadava P., Kumar K., Singh I., Kaul T., Pattanayak A., Agrawal P.K. (2018). Insights into maize genome editing via CRISPR/Cas9. Physiol. Mol. Biol. Plants.

[55] Meers E., Van Slycken S., Adriaensen K., Ruttens A., Vangronsveld J., Du Laing G., Witters N., Thewys T., Tack F.M.G. (2010). The use of bio-energy crops (*Zea mays*) for ‘phytoattenuation’ of heavy metals on moderately contaminated soils: A field experiment. Chemosphere.

[56] Pandey V.C., Bajpai O., Singh N. (2016). Energy crops in sustainable phytoremediation. Renew. Sustain. Energy Rev..

[57] Peng R., Lin G., Li J. (2016). Potential pitfalls of CRISPR/Cas9-mediated genome editing. FEBS J..

